# Factors associated with chronic energy malnutrition among reproductive-age women in Ethiopia: An analysis of the 2016 Ethiopia demographic and health survey data

**DOI:** 10.1371/journal.pone.0243148

**Published:** 2020-12-11

**Authors:** Gizachew Worku Dagnew, Melash Belachew Asresie

**Affiliations:** Department of Reproductive Health and population studies, School of Public Health, College of and Health Science, Bahir Dar University, Bahir Dar, Ethiopia; University of Ghana, GHANA

## Abstract

**Background:**

Women with chronic-energy malnutrition persists in many developing countries, including Ethiopia. To avert this problem identifying the predictor variables for a high magnitude of underweight is paramount. Consequently, this study aimed to assess the factors associated with chronic energy malnutrition among reproductive-age women in Ethiopia.

**Methods:**

We used the 2016 Ethiopia demographic health survey data. The survey was a community-based cross-sectional study conducted from January 18 to June 27, 2016. A two-stage stratified cluster sampling technique was employed to select Participants. A total of 13,451 reproductive-age group women (age 15–49 years and who were not pregnant and < 2 months of postpartum) were included in the analysis. Both descriptive and analytical analyses were performed. A P-value of less than 0.05 was used to declare statistical significance.

**Results:**

About 22.6% (95%CI: 21.5%-23.6%) of reproductive-age women were underweight. The magnitude of underweight is highest in the Afar region (39.6%) and lowest in Addis Ababa city administration (13.5%). Women who lived in the rural area (AOR = 1.59; 95%CI: 1.19–2.12), those who did not attend formal education (AOR = 1.23; 95%CI: 1.01–1.50), unemployed women (AOR = 1.28; 95%CI:1.13–1.44), women who belong to the poorest household wealth index (AOR = 1.42; 95%CI:1.04–1.94), women who were not married (AOR = 1.41; 95%CI: 1.18–2.69), women who lived in Tigray and the pastoral regions have higher odds of underweight. On the other hand, women who lived in southern nations nationalities and people’s region, and women whose age group 25–34 years had lower odds of underweight.

**Conclusions:**

Chronic-energy malnutrition among reproductive-age women is high in Ethiopia. Improving the food security of rural, never married, and unemployed women would reduce the magnitude of underweight. Moreover, strengthening girls’ education, creating employment opportunities for women, and enhancing household income can further reduce the problem of chronic energy malnutrition.

## Introduction

Today the world population is affected by double burden malnutrition (over or undernutrition). Overnutrition refers to a problem of excessive and abnormal fat depositions in the body [1]. Undernutrition includes chronic energy and micronutrient deficiencies, and it remains a persistent problem for many developing regions around the world [2,3]. Maternal and child undernutrition causes 3.5 million deaths that account for one-third of the disease burden in children younger than 5 years, and 11% of total global disability-adjusted life-years (DALYs) [3]. Chronic energy malnutrition occurs when the level of energy intake is insufficient to meet the person’s energy requirement, assessed by using a body mass index (BMI) [4]. BMI is a simple index of weight-for-height (wt/ht^2^) that is used to classify adults’ nutrition levels [4,5].

Globally, In 2016, 170 million (9.4%) women age 15–49 years were underweight [6]. And it is dominant in the world’s poorest regions, especially in South Asia and Africa. In low- and middle-income countries (LMICs) the prevalence of underweight seen among reproductive-age women is up to ten times higher than in high- and upper-middle-income countries [6–8]. In Africa, 23.5% of reproductive-age women were underweight [9]. And about 16.6% of childbearing age women in Sub-Saharan Africa (SSA) have a low BMI secondary to chronic hunger [10,11].

Ethiopia is one of the most food-insecure countries in Africa. In 2015, Around 15 million people become food insecure [12]. According to the food and agriculture organization of the United Nations, food insecurity is “a situation that exists when people lack secure access to sufficient amounts of safe and nutritious food for normal growth and development and an active and healthy life” [13]. The combined effect of natural disasters like; El Nino related drought that affects harvesting livestock and inflation of food prices are contributing drivers increasing the vulnerability of food insecurity and undernutrition in Ethiopia [12,14]. As a result, in the 21^st^ century, many women are affected by severe or moderate nutrition deficiency [15,16]. According to the 2011 Ethiopian demographic and health survey (EDHS), 27% of childbearing age women had severe or moderate underweight, which is similar to the 2005 DHS data [17]. Small-scale studies in Ethiopia showed that a high number of underweight prevalence among reproductive-age women; which is 47.9% in Tigray [18], 48.6% in Ziway Dugda district [19], 41.1% in Afar [20], and 17.9% in Debretabor [21]. Living in food-insecure households, low consumption of dairy products, have low meal frequency, low education level, unimproved source drinking water, and living in rural residences are responsible for the high levels of women undernutrition [18–20,22,23].

Women with chronic-energy malnutrition have a higher risk of maternal mortality, morbidity, and disability-adjusted life years (DALYs). Most of them also face poor intellectual ability, low economic productivity, and problem during labor and delivery [2,3]. The nutrition of women also affects fetal birth outcomes [2,10]. Poor maternal nutrition increased the risk of miscarriage, preterm birth, low-birth weight, stillbirth, and overall growth and developmental delay. Especially, the first thousand days of a child's physical and neurological development highly affected by the women's nutrition level [23–25].

Ethiopia has set a national nutrition strategy since 2008 that focused on reducing child and women undernutrition [26]. However, different global and national study findings showed that the problem of undernutrition persists without any significant improvement [18,26–29]. As a result, the world health organization (WHO) recommended revising the national nutrition strategy for the next 2016–2020 [26]. Besides, identifying different factors that might contribute to women's high level of undernutrition is paramount important to avert the problem. Therefore, the purpose of this study aimed to assess the factors associated with chronic energy malnutrition among reproductive-age women (age 15–49 years and who were not pregnant and < 2 months of postpartum) in Ethiopia by employing the 2016 EDHS data.

## Materials and methods

### Data source

This analysis used the 2016 EDHS data, collected from January 18 to July 27, 2016. It was a community-based cross-sectional survey. A total sample of 15,683 women respondents included in the survey. The survey used a two-stage stratified cluster sampling procedure, which was represented at the regional and residence level. The survey was conducted among all 11 administrative regions found in Ethiopia. Initially, each region was stratified into urban and rural areas yielding 21 sampling strata. After stratification, a total of 645 enumeration areas (202 in urban areas and 443 in rural areas) were selected with probability proportional to enumeration are size based on the 2007 Ethiopia population and housing census. A household listing operation was done from September to December 2015. Then, from each cluster, 28 households were selected using a systematic random sampling technique [29].

**Fig 1:**
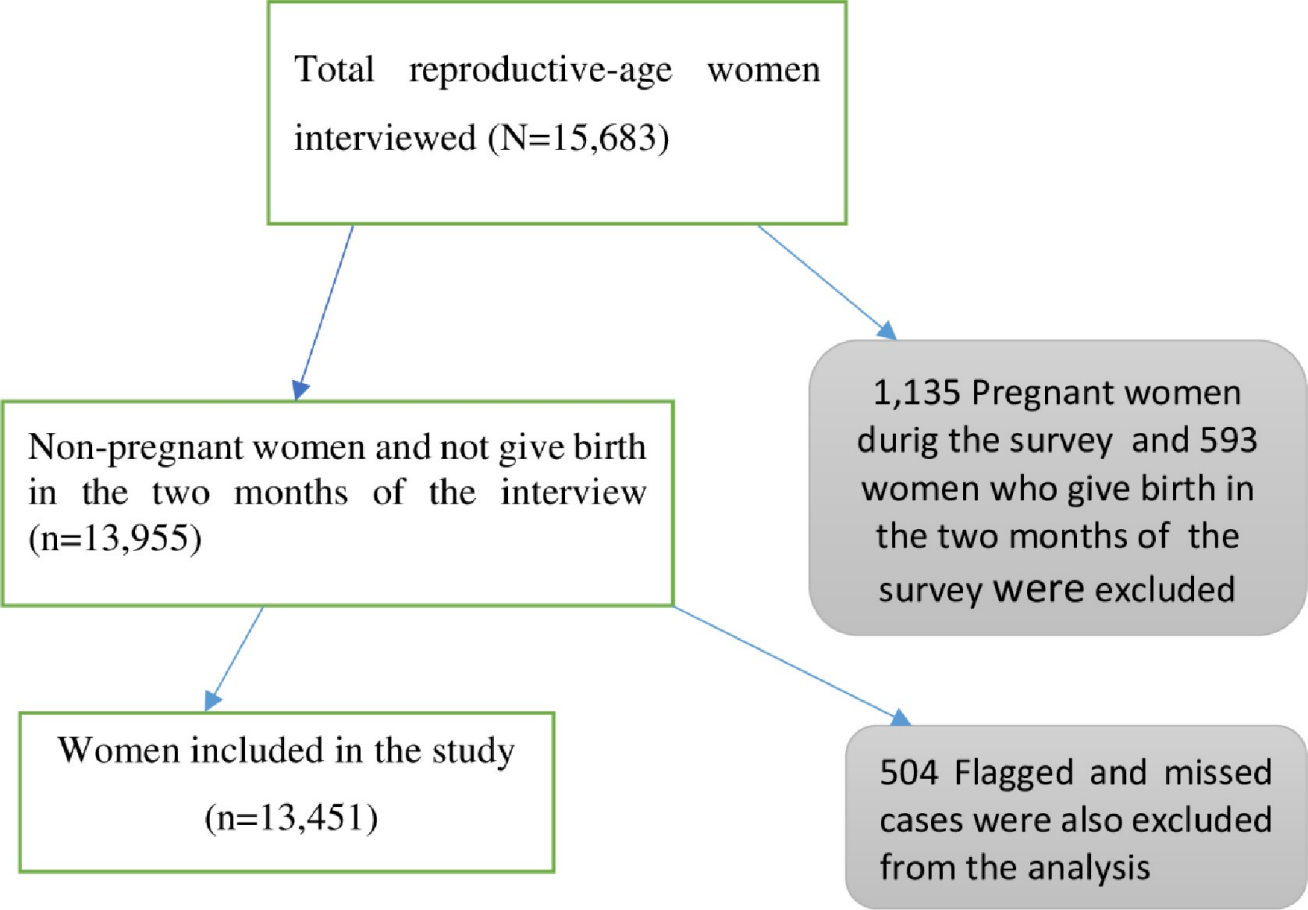
Schematic presentation showing a sampling of women with underweight in the 2016 EDH), Ethiopia.

This analysis included all women in the reproductive-age group (15–49 years) at the time of the interview. Women who were pregnant, postpartum (< 2 months of delivery), those who have outlier BMI values < 12.00 kg/m^2^ and > 50.00 kg/m^2^ (flagged cases), and missed cases were excluded from this study. Based on these criteria, a total of 13,451 reproductive-age women were included in the analysis **(Fig 1).**

### Measurements

#### Outcome variable

The outcome variable in this analysis was underweight among reproductive-age women (15–49 years); BMI is computed by weight in kilograms divided by height in meters squared. To determine the nutrition status of women, we implemented the WHO BMI cut-off points [30]. A woman whose BMI < 18.50 Kg/m^2^ was considered chronic energy malnutrition (underweight).

#### Independent variables

The independent variables included in this analysis were women age, residence (rural and urban), region, religion, education status, marital status, wealth index (poorest, poorer, middle richer, and richest), employment status (employed and unemployed), family size, history of alcohol intake, media exposure, sources of drinking water (Improved and unimproved), and nutrition counseling.

#### Frequency of media exposure

It was assessed the participants’ exposure to newspaper/magazine, radio, and/or television. It is coded as “not at all” when the women responded there is no media exposure, “less than once a week” when the women responded they have exposure for one or more types of the above-mentioned media, but for less than once a week, and “at least once a week” when the women responded they have exposure for one or more types of above-mentioned media for at least once a week.

#### Region

Recoded as; ‘the three Metropolis’ (which include Addis Ababa, Harari, and Dire Da’wa), Tigray, Amhara, Oromia, SNNPR, and “pastoral regions” (which include Afar, Benshangul-Gumuz, Gambela, and Somali)

### Statistical analysis

STATA version 14.0 was used to conduct the analysis. For examining prevalence and factors associated with underweight both descriptive and analytical analyses were computed. The data on women were weighted to account for sampling probability and non-response. Besides, the data were adjusted to account for the complex survey design and robust standard errors. Descriptive statistics were calculated for all the above-described variables. Cross-tabulation and chi-square statistics were conducted to show the urban to rural disparities of overweight/obesity by women’s background characteristics.

Bivariate logistics regression analysis was conducted to select the candidate variable for multivariable logistics regression. Variables with a p-value ≤ 0.2 in the binary logistic regression analysis were included in a multivariable logistic regression model. The multi-variable binary logistics regression analysis was done to show the explanatory variable independent effect for the outcome variable (overweight/obesity). The descriptive results are presented as proportions and the regression results are presented as adjusted odds ratios (AOR) with 95% confidence intervals. Model fitness was checked by using Hosmer Lemeshow's model good fit (p>0.05).

## Ethical approval

The 2016 EDHS protocol was reviewed and approved by the National Ethics Review Committee of the Federal Democratic Republic of Ethiopia, Ministry of Science and Technology, and the Institutional Review Board of ICF International. The STATA format data was downloaded from the DHS program with permission.

## Results

### Socio-demographic characteristics of the study participants

A total of 13,451 non-pregnant reproductive-age women participated in this study. The median age with the Interquartile range (IQR) of the study participants was 27.0 ± 15 years. More than one-third (39.3%) of women were aged ≤ 24 years. About 77% of women were living in rural resident. Regarding the participants' education, 6,351 (47.2%) women did not attend formal education. One-third (33.3%) of women were below middle wealth quantile. Regarding their marital status, 8,285 (61.6%) women were married. Almost half (48.6%) of women were unemployed. About 56% of the study participants have no media exposure (Table 1).

**Table 1:** Characteristics of non-pregnant reproductive-age women in Ethiopia; 2016 EDHS.

Variables (n = 13,451)	Categories	Frequency	Percent
Age in years	≤24	5,289	39.3
	25–34	4,342	32.2
	≥35	3,820	28.4
Residence	Urban	3,071	22.8
	Rural	10,380	77.2
Region	Tigray	994	7.4
	Amhara	3,340	24.8
	Oromia	4,749	35.3
	SNNPR	2,804	20.8
	Metropolis region	939	7.0
	The pastoral region	625	4.6
Wealth index	Poor	2,162	16.1
	Poorest	2,310	17.2
	Middle	2,606	19.4
	Rich	2,717	20.2
	Richest	3,655	27.2
Educational status	No education	6,351	47.2
	Primary	4,744	35.3
	Secondary or above	2,356	17.5
Marital status	Single	3,818	28.4
	Married	8,285	61.6
	Divorced/widowed	1,348	10.0
Religion	Orthodox	6,012	44.7
	Muslim	3,976	29.6
	Protestant	3,209	23.9
	Catholic	102	0.8
	Others	119	1.1
Working status	Not working	6,541	48.6
	Working	6,910	51.4
Sources of drinking water	Improved	8,803	65.4
	Unimproved	4,648	34.6
Family size	1–4	4,739	35.2
	5–7	6,095	45.3
	8+	2,617	19.5
Frequency of media exposure	Not at all	7,475	55.6
	Less than once a week	2,376	17.7
	At least once a week	3,600	26.8
Ever drink Alcohol	Yes	4,931	36.7
	No	8,520	63.3
Counseled about nutrition	Yes	2,586	19.2
	No	10,865	80.8
Have own lands	Yes	5,314	39.5
	No	8,137	60.5

### Chronic-energy malnutrition among reproductive-age women

**Fig 2:**
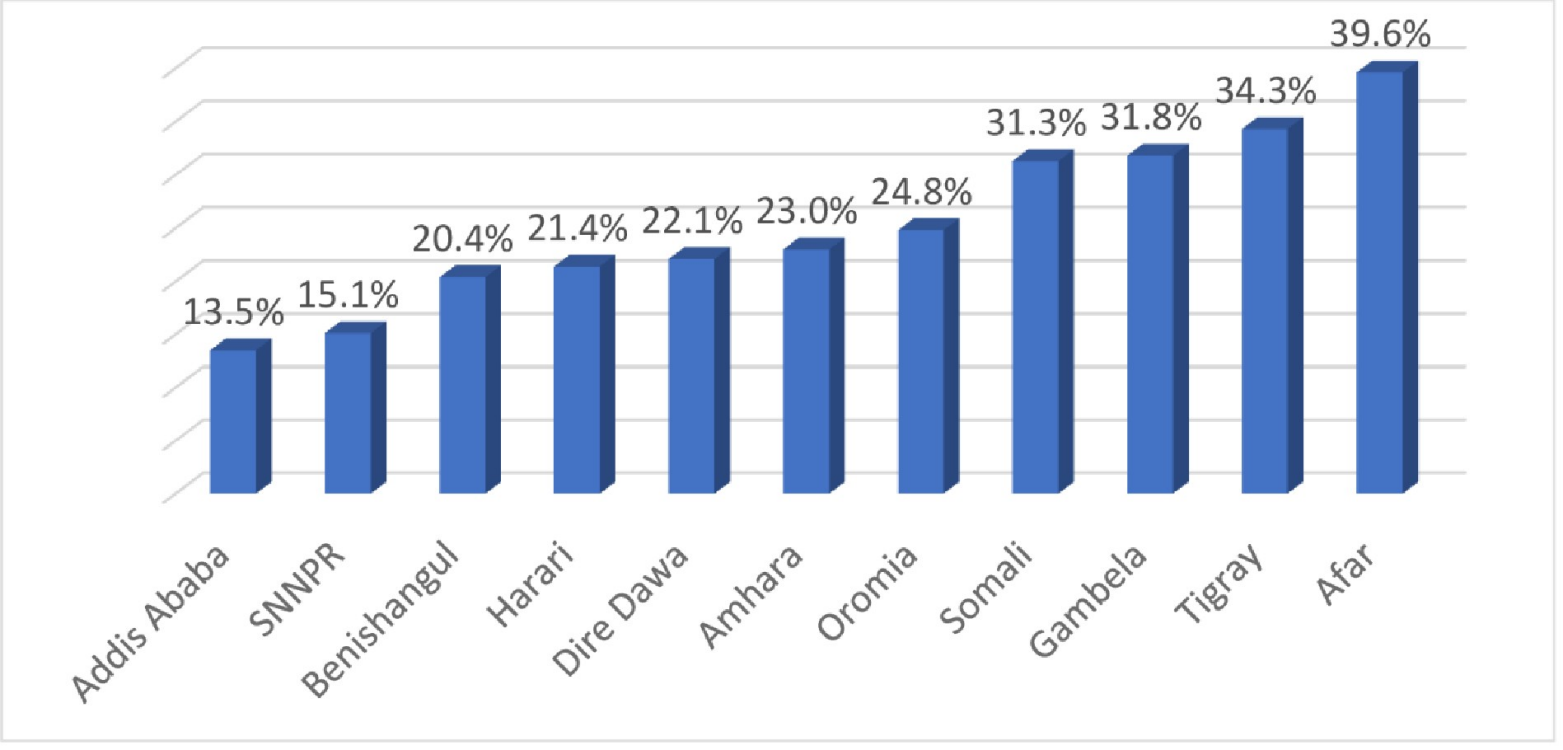
The magnitude of underweight among reproductive-age women by region; 2016 EDHS.

The analysis revealed that 3,033 non-pregnant and not-postpartum (< 2 months of delivery) reproductive-age group women (15–49 years) were underweight (P = 22.6%; 95%CI: 21.5%-23.6%). Of them, 913 (30.10%) women were severely thin (BMI < 17). The magnitude of underweight is highest in the Afar region (39.6%) followed by Tigray (34.3%), and lowest in Addis Ababa (13.5%) followed by SNNPR (15.1%) **(Fig 2).**

### Factors associated with Chronic energy malnutrition among reproductive-age women

On multivariable logistics regression; residence, women age, educational status, region, household wealth index, marital status, and history of alcohol intake have a statistically significant association with women underweight.

Women who lived in a rural area had higher odds of underweight compared to those who lived in the urban setting (AOR = 1.59; 95%CI: 1.19–2.12). Women who did not attend formal education had higher odds of underweight compared to those who attend secondary or above education (AOR = 1.23; 95%CI: 1.01–1.50). Women whose age group 25–34 years were 20% less likely to underweight compared to those who were age group of 15–24 years (AOR = 0.80; 95%CI: 0.67–0.94). Women who lived in the Tigray region (AOR = 2.01;95%CI: 1.56–2.58) and pastoral regions (AOR = 1.22; 95%CI: 1.01–1.47) had higher odds of underweight compared to those who lived in the Oromia region. On the other hand, women who lived in SNNPR had lower odds of underweight compared to the reference region Oromia (AOR = 0.50; 95%CI: 0.41–0.61). Women who have the poorest household wealth quantile had higher odds of underweight compared to women who have the richest wealth quantile. Single reproductive-age women had higher odds of underweight compared to women who were married (AOR = 1.41;95%CI: 1.18–1.69). Similarly, unemployed women had higher odds of underweight compared to their counterparts (AOR = 1.28; 95%CI: 1.13–1.44) (Table 2).

**Table 2:** Factors associated with chronic energy malnutrition among reproductive-age women in Ethiopia; 2016 EDHS.

Variables (n = 13,451)	Underweight		COR (95%CI)	AOR (95%CI)
	Yes	No		
**Residence**				
Rural	24.8	75.2	1.89 (1.62–2.22) ***	1.59 (1.19–2.12) **
Urban	14.8	85.2	1	1
**Educational status**				
No formal education	23.4	76.6	1.43 (1.21–1.70) ***	1.23 (1.01–1.50) *
Primary	23.8	76.2	1.47 (1.22–1.76) ***	1.22 (1.01–1.47) *
Secondary or above	17.6	82.4	1	1
**Age category**				
15–24	25.8	74.2	1.0	1
25–34	18.4	81.6	0.65 (0.57–0.74) ***	0.80(0.67–0.94) **
≥35	22.8	77.2	0.85 (0.74–0.98) *	1.02(0.85–1.23)
**Region**				
Oromia	24.8	75.2	1.0	1
Amhara	23.0	77.0	0.91 (0.76–1.08)	1.13(0.93–1.37)
SNNPR	15.1	84.9	0.54 (0.44–0.66) ***	0.50(0.41–0.61) ***
Tigray	34.3	65.7	1.58 (1.29–1.95) ***	2.01(1.56–2.58) ***
The metropolis city	14.4	85.6	0.51 (0.43–0.61) ***	0.98(0.77–1.25)
Pastoral regions	30.5	69.5	1.33 (1.13–1.57) **	1.22(1.01–1.47) *
**Marital status**				
Married	21.3	78.7	1.0	
Single	25.4	74.6	1.26 (1.12–1.42) ***	1.41(1.18–1.69) ***
Widowed/divorced	22.3	77.7	1.06 (0.88–1.28)	1.04(0.85–1.27)
**Wealth index**				
poorest	28.5	71.5	2.09 (1.70–2.57) ***	1.42(1.04–1.94) *
Poorer	23.4	76.6	1.60 (1.32–1.95) ***	1.15(0.85–1.56)
Middle	25.1	74.9	1.76 (1.44–2.14) ***	1.30(0.97–1.73)
Richer	23.5	76.5	1.61 (1.34–1.93) ***	1.20(0.91–1.59)
Richest	16.0	84.0	1	1
**Employment status**				
Unemployed	25.2	74.8	1.35 (1.21–1.50) ***	1.28(1.13–1.44) ***
Employed	20.0	80.0	1	1
**Have own land**				
Yes	21.4	78.6	1	1
No	23.3	76.7	1.12 (0.98–1.27)	1.16 (0.99–1.36)
**Source of drinking water**				
Improved	21.5	78.5	1	1
Unimproved	24.5	75.5	1.18 (1.04–1.35) *	1.00(0.87–1.14)
**Ever drinks alcohol**				
Yes	21.2	78.8	1	1
No	23.3	76.7	1.13 (0.99–1.29)	1.31(1.11–1.55) **
**Family size**				
1–4	21.8	78.2	1.0	1
5–7	22.3	77.7	1.03 (0.89–1.19)	0.94 (0.80–1.10)
8+	24.6	75.4	1.17 (0.97–1.42)	0.94(0.77–1.15)
**Got nutrition counseling**				
Yes	20.6	79.4	1.0	1
No	23	77	0.87 (0.74–1.02)	0.98(0.82–1.18)

*** p<0.001

** p<0.01

* p<0.05, COR: Crude Odd Ratio, AOR: Adjusted Odd Ratio, CI: Confidence Interval.

## Discussion

This study analyzed the national and regional prevalence of chronic energy malnutrition among reproductive-age women in Ethiopia. The determinants of underweight among reproductive-age women were also analyzed.

The findings of this study revealed that 22.6% of reproductive-age women were underweight. This finding was consistent with the findings of a multinational African study (23.5%) and India [9,31]. However, this was lower than the 2005 and 2011 national EDHS report, which is 27% [29,32]. This showed that the trend of undernutrition has some improvement in the country. The reason might be due to increased women's access to higher education and employment opportunity to generate income. The percentage of women with no education has decreased over the last decade, from 66% in 2005 to 47% in 2016. Similarly, the current finding is lower than studies done in Afar (41.1%), Tigray (47.9%), Ziway Dugda district Arsi Zone (48.6%) [18–20]. The reason for the low level of underweight in the current study compared to other studies could be the difference in the level of food security; Afar and Tigray are among the most food-insecure regions in Ethiopia [14]. The other possible reason could also be the difference in study setting; the current analysis used participants from both the rural and urban. Most studies showed that the magnitude of chronic energy malnutrition is high among rural women than urban [9,29]. Conversely, the current study finding is higher than the studies done in mainland Tanzania (11%), Kenya (12.2%) [33,34], and other studies from Ethiopia; Debretabor (17,9%), and Offa district Wolayita zone (15.8%) [21,35]. The reason for this variety might be the difference in the study setting, education, and marital status of the study participants. Educated and married women are less likely to be undernourished than their counterparts [9,36].

In this study, women who were living in rural areas had higher odds of undernutrition compared to those who lived in urban areas. Which is in line with other studies [9,37–39]. The possible explanation could be more urban women have higher education and employment than their rural counterparts. Educated women can understand the importance of adequate nutrition for health.

The findings of this study also revealed that underweight is significantly higher among non-educated women compared to those who have secondary or above education. Which was consistent with the study from Gondar Ethiopia, Kenya, India, and multi-national studies from Africa [9,31,34,38]. The first reason for this might be education; it can make a difference inwomen's employment. The less educated women have fewer employment opportunities than more educated women. The other reason might also be the variety of nutrition awareness level.

The magnitude of underweight had a significant association with women’s residence region. Women who were living in Tigray and the pastoral regions had higher odds of underweight compared to those who lived in the Oromia region. On the other hand, women who were living in SNNPR had lower odds of underweight compared to the reference region. This could be secondary to the difference in the levels of food security and the production of cereals in those regions.

In this study, unemployed women had higher odds of underweight compared to employed women. This was supported by studies done in Kenya, Myanmar [34,36]. This could be due to the difference in wealth. Most unemployed women have the lowest wealth quantile and they are a dependent population. In the current study, women who have the poorest household wealth quantile had higher odds of underweight compared to those who have the highest household wealth quantile. This is in line with other studies [9,33,40].

The other findings of this study revealed that married women had lower odds of underweight compared to those who were single. Similarly, women whose age group 25–34 years had lower odds of underweight compared to those who have 15–24 years of age. This finding is supported by other studies [33,36,40–42]. The first explanation for this might be different in nutrition requirements. Adolescents girls (age 15–24 years) are more vulnerable to undernutrition because they are growing faster than at any time, and they have high nutritional requirements to meet the physiological demand for development compared to adult women [43]. The second possible explanation might also be a higher proportion of women age above 24 years are married and have high childbirth history than single women. And most of those women have an income source by themselves or from their partners that afford to take excessive energy-dense meals during the postpartum period or else [44,45]. Additionally, in many cases, the households of never-married women have relatively high food insecurity than those of married women, making it more difficult for them to obtain sufficient food [46].

In this study, some important behavioral and clinical (HIV/AIDS and other chronic health conditions) factors that could explain the magnitude of underweight was not available. This includes diet diversity, frequency, and amount as well as type (nutritional history) and level of physical activity. Moreover, the causality of associations cannot be established because of the cross-sectional method employed in the DHS.

## Conclusions

This analysis revealed that the magnitude of the underweight among reproductive-age group women is high. Women's age, residence, educational status, marital status, employment, household wealth, and women's residence regions are the independent predictors of underweight. Working to improve the food security of women focusing on rural, unemployed, single, and adolescent (15–24 years) girls would reduce the burden of underweight. Moreover, strengthening girls’ education, households' economic productivity, and creating employment opportunities for women can further reduce the problem of malnutrition.

## Abbreviations

AOR: Adjusted Odds Ratio
BMI: Body Mass Index
CI: Confidence Interval
EDHS: Ethiopian Demographic and Health Survey
LMICs: Low and Middle-Income Countries
SNNPR: Southern Nations, Nationalities, and People’s Region
SSA: Sub-Saharan Africa
WHO: World Health Organization

## References

[1] World Health Organization. Global status report on noncommunicable diseases.: World Health Organization; 2014.

[2] Hoddinott J. The economic cost of malnutrition The Road to Good Nutrition: Karger Publishers; 2013 p. 64–73.

[3] Black RE, Allen LH, Bhutta ZA, Caulfield LE, De Onis M, Ezzati M, et al Maternal and child undernutrition: global and regional exposures and health consequences. The lancet. 2008;371(9608):243–60. 10.1016/S0140-6736(07)61690-0 18207566

[4] James W, Ferro-Luzzi A, Waterlow JC. Definition of chronic energy deficiency in adults. Report of a working party of the International Dietary Energy Consultative Group. European journal of clinical nutrition. 1988;42(12):969–81. 3148462

[5] World Health Organization. Global status report on noncommunicable diseases 2014: World Health Organization; 2014.

[6] Fanzo J, Hawkes C, Udomkesmalee E, Afshin A, Allemandi L, Assery O, et al 2018 Global Nutrition Report: Shining a light to spur action on nutrition. 2018.

[7] Abarca-Gómez L, Abdeen ZA, Hamid ZA, Abu-Rmeileh NM, Acosta-Cazares B, Acuin C, et al Worldwide trends in body-mass index, underweight, overweight, and obesity from 1975 to 2016: a pooled analysis of 2416 population-based measurement studies in 128· 9 million children, adolescents, and adults. The Lancet. 2017;390(10113):2627–42. 10.1016/S0140-6736(17)32129-3 PMC5735219 29029897

[8] Abdullah A. The double burden of undernutrition and overnutrition in developing countries: an update. Current obesity reports. 2015;4(3):337–49. 10.1007/s13679-015-0170-y 26627492

[9] Desyibelew HD, Dadi AF. Burden and determinants of malnutrition among pregnant women in Africa: A systematic review and meta-analysis. PloS one. 2019;14(9). 10.1371/journal.pone.0221712 31490956 PMC6730925

[10] Razak F, Finlay JE, Subramanian S. Maternal underweight and child growth and development. The Lancet. 2013;381(9867):626–7. 10.1016/S0140-6736(13)60344-X 23439098

[11] Lartey A. Maternal and child nutrition in Sub-Saharan Africa: challenges and interventions. Proceedings of the Nutrition Society. 2008;67(1):105–8. 10.1017/S0029665108006083 18234138

[12] Rashid S, Dorosh P, Alemu D. Grain markets, disaster management, and public stocks: Lessons from Ethiopia. Global Food Security. 2018;19:31–9.

[13] Napoli M, De Muro P, Mazziotta M. Towards food insecurity Multidimensional Index (FIMI). Master in Human Development and Food Security. 2011:1–72.

[14] Prášková DM. The 2015–2016 famine threat in Ethiopia: a study of the relevance of famine archetypes. AUC Geographica. 2018;53(2):193–206.

[15] World Health Organization. Accelerating nutrition improvements (ANI): mapping of stakeholders and nutrition actions in three scaling-up countries in sub-Saharan Africa: report of a meeting, 27–28 February 2014, Addis Ababa, Ethiopia. 2014.

[16] Bossuyt A. Moving toward nutrition-sensitive agriculture strategies and programming in Ethiopia. Agriculture for Improved Nutrition: Seizing the Momentum. 2019:165.

[17] Abrha S, Shiferaw S, Ahmed KY. Overweight and obesity and its socio-demographic correlate among urban Ethiopian women: evidence from the 2011 EDHS. BMC Public Health. 2016;16(1):636. 27457223 10.1186/s12889-016-3315-3 PMC4960736

[18] Abraham S, Miruts G, Shumye A. Magnitude of chronic energy deficiency and its associated factors among women of reproductive age in the Kunama population, Tigray, Ethiopia, in 2014. BMC nutrition. 2015;1(1):12.

[19] Ferede A, Lemessa F, Tafa M, Sisay S. The prevalence of malnutrition and its associated risk factors among women of reproductive age in Ziway Dugda District, Arsi Zone, Oromia Regional State, Ethiopia. public health. 2017;152:1–8. 10.1016/j.puhe.2017.06.011 28715656

[20] Abdu J, Kahssay M, Gebremedhin M. Household food insecurity, underweight status, and associated characteristics among women of the reproductive age group in Assayita District, Afar Regional State, Ethiopia. Journal of environmental and public health. 2018;2018. 10.1155/2018/7659204 PMC5977031 29887896

[21] Engidaw MT, Gebremariam AD, Tiruneh SA, Asnakew DT, Abate BA. Chronic Energy Deficiency and its Associated Factors Among Lactating Women in Debre Tabor General Hospital, Northcentral Ethiopia. Journal of Family Medicine and Health Care. 2019;5(1):1–7.

[22] Regassa N, Stoecker BJ. Contextual risk factors for maternal malnutrition in a food-insecure zone in southern Ethiopia. Journal of biosocial science. 2012;44(5):537–48. 10.1017/S002193201200017X 22716940

[23] Ahmed T, Hossain M, Sanin KI. Global burden of maternal and child undernutrition and micronutrient deficiencies. Annals of Nutrition and Metabolism. 2012;61(Suppl. 1):8–17. 10.1159/000345165 23343943

[24] Dewey KG, Begum K. Long‐term consequences of stunting in early life. Maternal & child nutrition. 2011;7:5–18. 10.1111/j.1740-8709.2011.00349.x 21929633 PMC6860846

[25] Imdad A, Bhutta ZA. Maternal nutrition and birth outcomes: Effect of balanced protein‐energy supplementation. Pediatric and perinatal epidemiology. 2012;26:178–90. 10.1111/j.1365-3016.2012.01308.x 22742610

[26] World Health Organization. Accelerating Nutrition Improvements (ANI): mapping of stakeholders and nutrition actions in three scaling-up countries in sub-Saharan Africa: report of the second meeting, 10 February 2015, Kampala, Uganda. 2016.

[27] Bossuyt A. Moving toward nutrition-sensitive agriculture strategies and programming in Ethiopia. Agriculture for Improved Nutrition: Seizing the Momentum. 2019;165.

[28] Birhane T, Shiferaw S, Hagos S, Mohindra KS. Urban food insecurity in the context of high food prices: a community-based cross-sectional study in Addis Ababa, Ethiopia. BMC public health. 2014;14(1):680 10.1186/1471-2458-14-680 24993286 PMC4227136

[29] Central Statistical Agency (CSA) [Ethiopia] and ICF. Ethiopia Demographic and Health Survey 2016. Addis Ababa, Ethiopia, and Rockville, Maryland, USA: CSA and ICF. 2016.

[30] World Health Organization. Obesity: preventing and managing the global epidemic: World Health Organization; 2000. 11234459

[31] Devgun P, Mahajan SL, Gill KP. Prevalence of chronic energy deficiency and socio-demographic profile of women in slums of Amritsar city, Punjab, India. Science International Journal of Research in Health. 2014;2(2):527–32.

[32] Central Statistical Agency [Ethiopia] and ORC Macro. Ethiopia Demographic and Health Survey 2005. Addis Ababa, Ethiopia and Calverton, Maryland, USA: Central Statistical Agency, and ORC Macro 2006.

[33] Mtumwa AH, Paul E, Vuai SA. Determinants of undernutrition among women of reproductive age in Tanzania mainland. South African Journal of Clinical Nutrition. 2016;29(2):75–81.

[34] Mbagaya G, Kotut J, Wafula S, Ettyang G. Protein-energy malnutrition among women of childbearing age in semi-arid areas of Keiyo District, Kenya. 2014.

[35] Julla BW, Haile A, Ayana G, Eshetu S, Kuche D, Asefa T. Chronic Energy Deficiency and Associated Factors among Lactating Mothers (15–49 years old) in Offa Woreda, Wolayita Zone, SNNPRs, Ethiopia. World Scientific Research. 2018;5(1):13–23.

[36] Hong SA, Peltzer K, Lwin KT, Aung LS. The prevalence of underweight, overweight, and obesity and their related socio-demographic and lifestyle factors among adult women in Myanmar, 2015–16. PloS one. 2018;13(3):e0194454 10.1371/journal.pone.0194454 29547655 PMC5856399

[37] Tebekaw Y. Women’s decision-making autonomy and their nutritional status in Ethiopia: socio-cultural linking of two MDGs The demographic transition and development in Africa: Springer; 2011 p. 105–24.

[38] Kumera G, Gedle D, Alebel A, Feyera F, Eshetie S. Undernutrition and its association with socio-demographic, anemia, and intestinal parasitic infection among pregnant women attending antenatal care at the University of Gondar Hospital, Northwest Ethiopia. Maternal health, neonatology, and perinatology. 2018;4(1):18. 10.1186/s40748-018-0087-z PMC6134711 30214818

[39] Kamal SM, Hassan CH, Alam GM. Dual burden of underweight and overweight among women in Bangladesh: patterns, prevalence, and sociodemographic correlates. Journal of health, population, and nutrition. 2015;33(1):92 25995726 PMC4438653

[40] Al Kibria GM, Swasey K, Hasan MZ, Sharmeen A, Day B. Prevalence, and factors associated with underweight, overweight, and obesity among women of reproductive age in India. Global Health Research and Policy. 2019;4(1):24. 31517064 10.1186/s41256-019-0117-z PMC6729094

[41] Hashan MR, Das Gupta R, Day B, Al Kibria GM. Differences in prevalence and associated factors of underweight and overweight/obesity according to rural-urban residence strata among women of reproductive age in Bangladesh: evidence from a cross-sectional national survey. BMJ Open. 2020;10(2):e034321 10.1136/bmjopen-2019-034321 32024791 PMC7045126

[42] Tanwi TS, Chakrabarty S, Hasanuzzaman S. Double burden of malnutrition among ever-married women in Bangladesh: a pooled analysis. BMC Women's Health. 2019;19(1):24 10.1186/s12905-019-0725-2 30704454 PMC6357418

[43] Chaparro C, Lutter C. Underweight, short stature, and overweight in adolescents and young women in Latin America and the Caribbean. Healthy Life Course, Pan America Health Organization. 2011:12.

[44] Steyn NP, Mchiza ZJ. Obesity and the nutrition transition in Sub‐Saharan Africa. Annals of the New York Academy of Sciences. 2014;1311(1):88–101. 10.1111/nyas.12433 24725148

[45] Lopez‐Arana S, Burdorf A, Avendano M. Trends in overweight by educational level in 33 low‐and middle‐income countries: the role of parity, age at first birth, and breastfeeding. Obesity Reviews. 2013;14(10):806–17. 10.1111/obr.12051 23782957 PMC3804307

[46] Franklin B, Jones A, Love D, Puckett S, Macklin J, White-Means S. Exploring mediators of food insecurity and obesity: a review of recent literature. Journal of community health. 2012;37(1):253–64. 10.1007/s10900-011-9420-4 21644024 PMC3334290

